# Poor Quality in Systematic Reviews on PTSD and EMDR – An Examination of Search Methodology and Reporting

**DOI:** 10.3389/fpsyg.2019.01558

**Published:** 2019-07-09

**Authors:** Elin Opheim, Per Normann Andersen, Marianne Jakobsen, Bjørn Aasen, Kari Kvaal

**Affiliations:** ^1^Inland Norway University of Applied Sciences, Elverum, Norway; ^2^Norwegian Centre for Violence and Traumatic Stress Studies, Oslo, Norway; ^3^EMDR Norway, Oslo, Norway

**Keywords:** systematic reviews, Cochrane handbook, PRISMA, search methodology, critical appraisal

## Abstract

**Background:** Different user groups regard systematic reviews as reliable and valuable sources for answering research questions. For systematic reviews to fulfill their purpose, methodological quality in all stages are of importance. The studies identified in a systematic search form the basis of the review, thus the search process methodology is important for both performing and reporting the search. The purpose of the present study was to evaluate the quality of non-Cochrane systematic reviews by analyzing how they perform and report the search. This is exemplified by systematic reviews on eye movement desensitization and reprocessing (EMDR), a trauma-focused therapy commonly used for post-traumatic stress disorder (PTSD).

**Methods and Results:** We examined the method chapters of 20 systematic reviews on the subject, and rated their searches and reporting using relevant elements from the Cochrane Handbook and PRISMA. We found inadequacies in the methods employed for searching and reporting the search strategy, which could have been avoided by greater adherence to guiding documents for performing systematic reviews.

**Conclusion:** Our findings raise important questions for future debate on the risk of omitting studies, thus impairing the conclusions in a systematic review. For clinical purposes, researchers should investigate if, and how, the search strategy in a systematic review affects the body of knowledge and the results.

## Introduction

Moher et al. (2007) estimated a yearly production of 2,500 systematic reviews, equaling a daily production of 6.8. By 2014 the daily production had increased to 22 (Page et al., 2016), leading to the assumption that the annual publication of systematic reviews is greater than that of randomized controlled trials (Ioannidis, 2016). The need for systematic reviews is not disputed, as they define the state of knowledge and provide a synthesis of previous research on a given issue (Cooper, 1982; Foster and Jewell, 2017), are valuable for researchers, practitioners, consumers, and policy makers (PLoS Medicine Editors, 2007; Liberati et al., 2009), inform future research (Liberati et al., 2009; Ioannidis, 2016), and help practitioners remain up-to-date in a time efficient way (Moher et al., 2007; Schlosser et al., 2007; Liberati et al., 2009; Wormald and Evans, 2018).

The Cochrane handbook provides the following explanation of what constitutes a systematic review: “A systematic review attempts to collate all empirical evidence that fits pre-specified eligibility criteria in order to answer a specific research question. It uses explicit, systematic methods that are selected with a view to minimizing bias, thus providing more reliable findings from which conclusions can be drawn and decisions made” (Higgins and Green, 2011). This definition distinguishes systematic reviews from other reviews, where the methodology is not explicitly stated, and the reader cannot replicate the process (Foster and Jewell, 2017).

Many systematic reviews include meta-analyses to provide estimates of effect, using statistical methods to combine data from all relevant studies (Higgins and Green, 2011). Thus, meta-analyses share recommendations for rigor, transparency, and reporting of methods with systematic reviews (Morton et al., 2018).

The time and effort required to conduct a high-quality systematic review are considerable, so it is essential to justify the need for a systematic review or meta-analysis (Wormald and Evans, 2018), avoid redundancy (Ioannidis, 2016; Cooper et al., 2018), and ensure that it is trustworthy. Authors planning to conduct systematic reviews have good sources to guide them in methodological questions, such as The Cochrane handbook (Higgins and Green, 2011), Standards for systematic reviews from the Institute of Medicine (Institute of medicine, 2011), and Methods Guide for Effectiveness and Comparative Effectiveness Reviews from the Agency for healthcare research and quality (Agency for healthcare research and quality, 2015). There are also guiding documents for critical appraisal and reporting questions for systematic reviews such as: A MeaSurement Tool to Assess systematic Reviews (AMSTAR 2) (Shea et al., 2017), and Preferred Reporting Items for Systematic Reviews and Meta-Analyses (PRISMA) (Liberati et al., 2009). In addition, those new to critical appraisal can learn from systematic review checklists, such as the Critical Appraisal Skills Programme (CASP) checklist (Critical Appraisal, and Skills Programme., 2018).

The vast increase in the publication of systematic reviews can partially be explained by the following factors: for clinicians, systematic reviews can guide choices between different interventions (Page et al., 2016); systematic reviews are considered to be reliable and valuable (Bastian et al., 2010; Page et al., 2016); the authors do not need to apply for approval from an ethics committee (Wormald and Evans, 2018); and several institutions place a great deal of emphasis on the number of publications, which may drive authors to conduct and publish systematic reviews that might lack the necessary systematic rigor (Page et al., 2016). We also recognize that financing can be an issue in research projects, one of many barriers that do not apply to systematic reviews (Wormald and Evans, 2018).

The studies identified in a systematic search form the basis of the review, and the search process is of importance for the methodology for both performing and reporting the search (Liberati et al., 2009; Sampson et al., 2009; McGowan et al., 2016; Wormald and Evans, 2018). The systematic search process consists of deciding what sources to search, planning the search process, designing search strategies, managing references, and documenting and reporting the search process (Higgins and Green, 2011). Cooper et al. (2018) have identified eight key stages of the search process from central guidance documents for systematic reviews, as shown in Table 1.

**TABLE 1:** Key stages of the literature search process.

1. Deciding who should undertake the literature search
2. Determining the aim and purpose of a literature search
3. Preparing for the literature search
4. Designing the search strategy
5. Determining the process of literature searching and deciding where to search (bibliographic database searching)
6. Determining the process of literature searching and deciding where to search (supplementary search methods)
7. Managing the references
8. Documenting the search

*From Cooper et al. (2018).*

This process aims to build a sound base for analyzing and synthesizing evidence, thus minimizing the risk of not including all relevant studies (Cooper et al., 2018).

Controlled vocabulary, or subject terms, is considered central for retrieving relevant records (Higgins and Green, 2011). The use of relevant subject terms in a search strategy is one of six elements that highly impact the precision of the search and the number of relevant records retrieved (Sampson et al., 2009), other elements being translation of the research question, logical operator errors, line number errors and spelling errors. Subject specific databases use controlled vocabulary to index and describe the content of an article. Medical Subject Headings (MeSH^®^) used by, e.g., Medline, Pubmed, and Cochrane might be the most commonly known^1^. Other subject specific databases, such as PsycInfo, have their own controlled vocabulary. The controlled vocabulary helps the searcher by mapping synonyms and concepts that might differ in American English and British English, or where the same concept has been described in different words (Higgins and Green, 2011). Another advantage is the possibility to simultaneously search for more specific terms, ‘explode’ (Higgins and Green, 2011). Author guidelines may require authors to use MeSH-terms to describe the content of their article, rather than keywords chosen by the author, and the National Library of Medicine provides authors with tools for doing this (National Library of Medicine, 2017).

For systematic reviews to fulfill their purpose, methodological quality in all stages are of importance. Discussions on methodology are ongoing; we need for instance to discuss the term “systematic” and how systematic reviews and narrative reviews together can broaden our understanding of a topic (Greenhalgh et al., 2018). Reporting has been found to be inconsistent (Moher et al., 2007; Koffel and Rethlefsen, 2016; Page et al., 2016), so not all systematic reviews are systematic.

The purpose of the present study was to evaluate the quality of non-Cochrane systematic reviews by analyzing their reporting methods, more specifically how the search to identify a body of evidence was performed. This is exemplified by systematic reviews on eye movement desensitization and reprocessing (EMDR), a trauma-focused therapy commonly used for post-traumatic stress disorder (PTSD; Bisson et al., 2013). We carried out a scoping search to map existing research on EMDR, and found a large number of systematic reviews and meta-analyses on PTSD. Cochrane published systematic reviews on psychological therapies for PTSD in adults in 2007 and 2013 (Bisson and Andrew, 2007; Bisson et al., 2013), and for children and adolescents in 2012 and 2016 (Gillies et al., 2012, 2016).

## Materials and Methods

**FIGURE 1:**
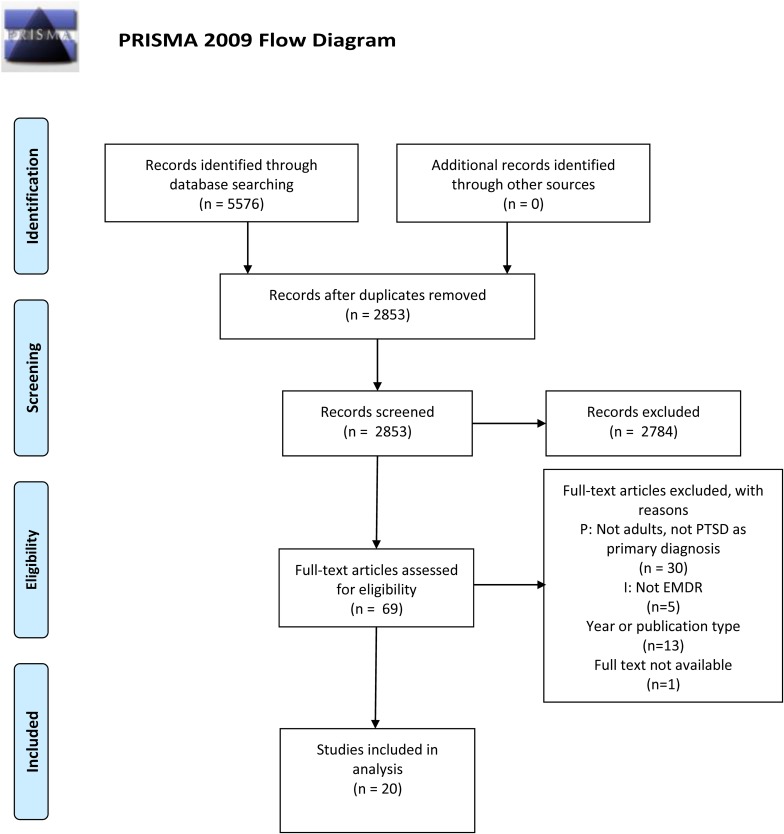
Prisma2009 flow diagram. Adapted from Moher et al. (2009).

We performed a systematic search for studies on EMDR using the following subject term: Eye movement desensitization therapy, and text words: emdr; eye movement desensiti?ation. The search strategy was peer reviewed and adapted to the different databases. A list of the databases and the search strategy is available from http://hdl.handle.net/11250/2597407. The search started on December 12th 2017 and ended on January 25th 2018. In total, 5,576 studies were retrieved. Duplicates (*N* = 2,723) were removed using EndNote X8 (Clarivate Analytics). Systematic reviews and meta-analyses reporting the effects of EMDR were manually sorted into a separate EndNote-group, after which those covering PTSD in adults were added to a subgroup, resulting in 22 systematic reviews and meta-analyses. We limited the inclusion of systematic reviews and meta-analyses to those published between 2010 and 2017, as we assumed that the authors would be familiar with the PRISMA guidelines, AMSTAR or Cochrane Handbook from 2010. We have excluded one review as we were unable to obtain it in full text (Novo Navarro et al., 2016), and one Cochrane review due to the aim of the study (Bisson et al., 2013). See Figure 1.

We initially examined all studies independently using the following broad question regarding the literature search from the Norwegian version of the CASP checklist for Systematic reviews (Critical Appraisal, and Skills Programme., 2018): “Do you think all the important, relevant studies were included?”. Response alternatives are Yes, Unclear or No. This question corresponds with that in AMSTAR 2, asking: “Did the review authors use a comprehensive literature search strategy?” (Shea et al., 2017). Secondly, disagreements were solved through discussion. Afterward, the methods chapters were examined and the searches and reporting rated using relevant elements from the Cochrane Handbook (Higgins and Green, 2011) and PRISMA 2009 Checklist (Liberati et al., 2009).

Medline, Embase, and Central are regarded as the most important sources to search for trials (Higgins and Green, 2011). In addition, the Cochrane handbook suggests the inclusion of subject specific databases. PsycInfo and PILOTS (Published International Literature on Traumatic Stress) are relevant due to the research question of the systematic reviews. Reviews stating that these databases were used, were awarded one point for each database. If no database was mentioned the rating was zero.

A comprehensive search should consist of a combination of subject terms and text words (Higgins and Green, 2011), and we assumed that authors of systematic reviews using subject terms would provide precise information on this aspect. We considered the use of subject terms to be a more objective measure, than evaluating the text words used in the search strategy. Reviews that reported using subject terms received one point, reviews not using subject terms received zero points.

We have rated item 1 (Title), item 7 (Information sources), and item 8 (Search) from the PRISMA Checklist. We have also checked whether the reviews include a flow diagram (item 17), and whether or not they refer to the Cochrane review on psychological therapies for PTSD in adults (Bisson and Andrew, 2007; Bisson et al., 2013). These elements are rated with Yes when information on a given criterion is stated, and No when information is lacking, see Table 2.

**TABLE 2:** Elements used for rating search methods and reporting.

	**Item**	**Reported using/not using**
**Cochrane Handbook (search)**		
- Databases (minimum)	CENTRAL	1/0
	MEDLINE	1/0
	EMBASE	1/0
- Subject specific databases	PsycInfo	1/0
	PILOTS	1/0
- Controlled vocabulary		1/0
**PRISMA (reporting)**		
- **Title #1**	Title	Yes/No
- **Information sources #7**	Database	Yes/No
	Provider	Yes/No
	Dates of coverage	Yes/No
	Date last searched	Yes/No
- **Search #8**	Search strategy for one database	Yes/No
- **Study selection #17**	Flow diagram	Yes/No
**Context**	Reference to Cochrane review	Yes/No

## Results

The systematic reviews in Table 3 constitute our data sample.

**TABLE 3:** Included systematic reviews.

**Author**	**Year**
Chen et al.	2015
Chen et al.	2014
Cusack et al.	2016
Ehring et al.	2014
Erford et al.	2016
Goodson et al.	2011
Haagen et al.	2015
Haugen et al.	2012
Ho and Lee	2012
Jong et al.	2014
Lapp et al.	2010
Lenz et al.	2017
Mello et al.	2013
Stergiopoulos et al.	2011
Swan et al.	2017
Thomaes et al.	2014
Torchalla and Strehlau	2017
Tribe et al.	2017
Watts et al.	2013
Zantvoord et al.	2013

Eight reviews were published between 2010 and 2013; twelve reviews were published from 2014 to 2017.

Geographically, correspondence address for the included reviews is United States-6, The Netherlands-4, Great Britain-2, Canada-2, Australia-1, Brazil-1, Taiwan-1, China-1, Germany-1, and France-1.

The systematic reviews and meta-analyses were published in sixteen different journals, from ten different publishers.

### Initial Screening of Search Methods

The authors of three reviews (Stergiopoulos et al., 2011; Chen et al., 2014; Thomaes et al., 2014) had reported their search strategies in such a manner that we could answer the critical appraisal question: “Do you think all the important, relevant studies were included?” with “Yes”. For four reviews (Zantvoord et al., 2013; Cusack et al., 2016; Swan et al., 2017; Torchalla and Strehlau, 2017), it was unclear whether the reported search strategy had found all important, relevant studies; for two of these, Cusack et al. (2016) and Zantvoord et al. (2013), we were unable to access the full search strategy. We answered the question with “No” for thirteen of the reviews (Lapp et al., 2010; Goodson et al., 2011; Haugen et al., 2012; Ho and Lee, 2012; Mello et al., 2013; Watts et al., 2013; Ehring et al., 2014; Jong et al., 2014; Chen et al., 2015; Haagen et al., 2015; Erford et al., 2016; Lenz et al., 2017; Tribe et al., 2017).

### Databases and Subject Terms

**FIGURE 2:**
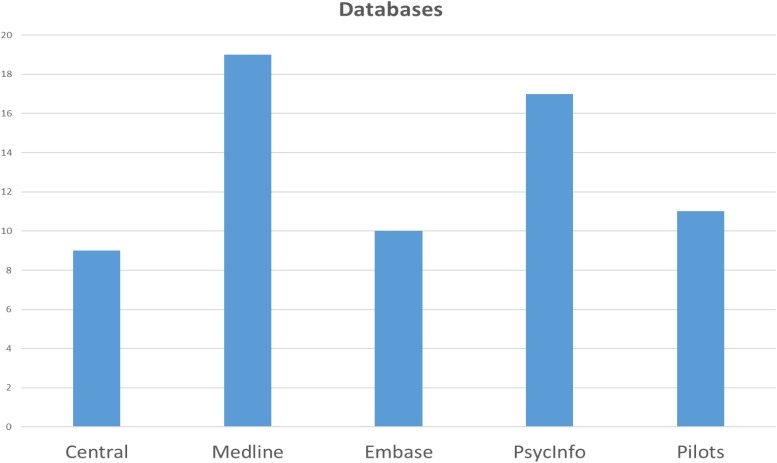
Number of reviews using selected databases.

Of the three databases (Central, Medline, Embase) recommended for all systematic reviews by the Cochrane handbook (Higgins and Green, 2011), only Medline/Pubmed was used by nineteen reviews. Ten reviews used Embase, and nine Central for identifying single studies. Seventeen reviews used PsycInfo, one review only used PsycInfo out of these databases, while three reviews used Medline, but not PsycInfo. Eleven studies in our sample used PILOTS. Five reviews reported using all five databases, see Figure 2. Four reviews reported using subject terms as part of their search strategy.

The scores for search characteristics in Table 4 are consistent with the findings from the initial screening of search methods, where three reviews were rated Yes, 4 Unclear, and 11 No. All the reviews rated between 1 and 3 were assessed as No, meaning we consider it unlikely that all important and relevant studies were found. The reviews receiving 6 points, were rated Yes [1] and Unclear [1]. One of the reviews scoring 4 points, was also rated yes in the initial screening.

**TABLE 4:** Scores for search characteristics.

1 point	1 SR	50%
2 points	6 SRs	
3 points	3 SRs	
4 points	4 SRs	40%
5 points	4 SRs	
6 points	2 SR	10%

*SR, systematic review. Higher score indicate better search characteristics.*

The total scores for the search, reporting and context are shown in Table 5.

**TABLE 5:** Included systematic reviews and meta-analysis with results for the search, reporting and context.

			**Cochrane Handbook (search)^2^**							**PRISMA (reporting)^3^**								
											**#7 Information sources**							
**Author:**	**Year:**	**Initial exami-nation^1^**	**Central**	**Medline**	**Embase**	**PsycInfo**	**Pilots**	**Subject terms**	**Sum:**	**#1 Title**	**Data-base**	**Provider**	**Coverage**	**Date last searched**	**Suppl. Techniques**	**#8 Search**	**#17 Flow diagram**	**Context**
Chen, L	2015	No	1	1	0	0	0	0	**2**	Yes	Yes	No	No^*^	No	Yes	Yes	No	Yes
Chen, Y.R.	2014	Yes	1	1	1	1	0	1	**5**	Yes	Yes	No	No^*^	No	Yes	No	Yes	No
Cusack, K	2016	Unclear	1	1	1	1	1	1	**6**	Yes	Yes	No	No^*^	Yes	Yes	Yes	Yes	No
Ehring, T	2014	No	1	1	0	1	1	0	**4**	Yes	Yes	No	No^*^	Yes	Yes	No	Yes	Yes
Erford, B. T.	2016	No	0	1	0	1	0	0	**2**	Yes	Yes	No	No^*^	No	Yes	No	Yes	Yes
Goodson, J.	2011	No	0	1	0	1	1	0	**3**	Yes	Yes	No	No	No	Yes	No	No	Yes
Haagen, J. F. G.	2015	No	0	1	1	1	1	0	**4**	Yes	Yes	Yes	No	No	No	No	Yes	Yes
Haugen, P.T.	2012	No	1	1	1	1	1	0	**5**	Yes	Yes	No	No	Yes	Yes	No	Yes	Yes
Ho, M	2012	No	0	1	0	1	0	0	**2**	No	Yes	Yes	No	No	Yes	No	No	Yes
Jong, K.	2014	No	0	1	0	1	0	0	**2**	Yes	Yes	No	No	Yes	Yes	No	Yes	No
Lapp, L. K.	2010	No	0	1	0	0	1	0	**2**	No	Yes	No	No	Yes	Yes	No	No	No
Lenz, A. S.	2017	No	0	0	0	1	0	0	**1**	Yes	Yes	No	No^*^	No	Yes	No	Yes	No
Mello, P.	2013	No	0	1	1	0	0	0	**2**	No	Yes	No	No^*^	Yes	No	No	No	Yes
Stergiopoulos, E.	2011	Yes	0	1	1	1	0	1	**4**	Yes	Yes	No	No	Yes	No	Yes	Yes	Yes
Swan, S	2017	Unclear	0	1	1	1	0	0	**3**	Yes	Yes	No	No	No	Yes	No	Yes	Yes
Thomaes, K	2014	Yes	1	1	1	1	1	1	**6**	Yes	Yes	No	No	Yes	Yes	Yes	No	Yes
Torchalla, I.	2017	Unclear	1	1	1	1	1	0	**5**	Yes	Yes	No	No	Yes	Yes	No	Yes	No
Tribe, R.H.	2017	No	0	1	0	1	1	0	**3**	Yes	Yes	Yes	No	Yes	Yes	No	Yes	No
Watts, B. V.	2013	No	1	1	0	1	1	0	**4**	Yes	Yes	No	No^*^	Yes	Yes	No	Yes	Yes
Zantvoord, J. B.	2013	Unclear	1	1	1	1	1	0	**5**	Yes	Yes	No	No^*^	Yes	Yes	Yes	Yes	Yes
			9	19	10	17	11	4										

^1^Initial examination answering the question: Do you think all the important, relevant studies were included? ^2^If databases and controlled vocabulary, as suggested in Cochrane handbook, were applied. ^3^If reporting adhered to PRISMA guidelines. ^*^ = general information on time span is reported.

### Documentation of the Search and Results in Accordance With PRISMA, Figure 3

17 out of 20 reviews identified the paper as either a “meta-analysis” or systematic review’ in the title. All reviews listed the names of the databases used in the search, but only three stated the names of the database providers. Database hosts or full text resources from one publisher were listed as databases in several of the reviews.

**FIGURE 3:**
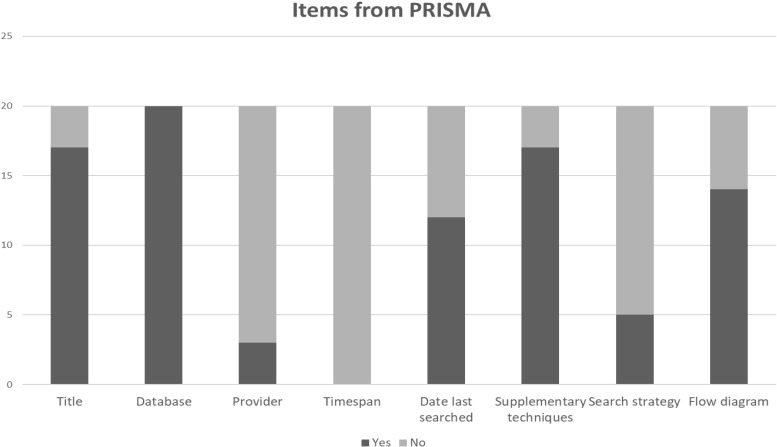
Use of PRISMA items. Number of articles reviewed adhering to PRISMA guidelines regarding the reporting of Title, Information sources, Search and Flow diagram.

Information on time span, and date on which the search ended was found in 9 and 12 reviews respectively. Time span searched is provided as general information, not for each database. Supplementary search techniques, such as contacting authors and manually searching journals, were used in 17 papers.

The full search strategy from at least one database was reported by five of the reviews.

A total of 14 reviews (70%) used a flow chart. Of these, only three used the PRISMA flowchart, referring to the PRISMA statement.

### Context

Seven of the reviews did not refer to the 2007 Cochrane systematic review on psychological therapies for PTSD in adults by Bisson, or the revised version in 2013 (Bisson and Andrew, 2007; Bisson et al., 2013).

## Discussion

Designing and conducting a systematic review or a meta-analysis is time-consuming if all the stages from planning to reporting are performed according to appropriate methodological standards (Liberati et al., 2009; Higgins and Green, 2011). The reported search methods can indicate the certainty that the synthesis or analysis is based on an accurate and complete evidence base (Sampson et al., 2009; Cooper et al., 2018).

Our initial screening found that only three out of the twenty systematic reviews or meta-analyses adhered to recommended systematic search methods. This is a general, overall evaluation of the search strategies reported, without applying specific criteria. In her study of 103 systematic reviews from prosthodontic and implant-related journals, Layton found that only 5% met all assessment criteria (Layton, 2017). A recent study of search strategies (Salvador-Olivan et al., 2019) found that 92.7% of 137 systematic reviews published in January 2018 contained strategies with error. It seems likely that our broad estimate of quality regarding the search is higher than that in studies using specific criteria.

High impact journals publish systematic reviews that have been found to be lacking in quality (Koffel and Rethlefsen, 2016), so there is a need for critical appraisal at article level (DORA, 2012).

The Cochrane handbook recommends that all systematic reviews perform searches in Central, Medline and Embase, with Central considered to be the best single database for searching for reports of trials (Higgins and Green, 2011). This recommendation seems to be unfamiliar to many of the authors in our sample. We do not consider the total number of databases to be relevant in our assessment. Many databases may look impressive to the reader, but to enhance comprehensiveness, it is important to search the appropriate databases, thus reducing the risk of database bias (Schlosser et al., 2007; Cooper et al., 2018). Access to appropriate databases can be a challenge if your institution does not subscribe, but PubMed and PILOTS are freely available, and the PILOTS database is central for studies on PTSD. It may be that authors lack knowledge about available and appropriate databases relevant to their research question.

All of the studies in our sample provided information on which databases they used, but only three named the providers of the databases. In contrast, Koffel and Rethlefsen (2016) found that 61% named the database provider. This information is important for the reproducibility of the search strategies; different providers give differing options and functionality for conducting a search (Cooper et al., 2018). Some of the authors seem to be confused about the difference between a database and a provider, stating that they have searched, e.g., Ebscohost and Medline. When only information on the provider is given, it is impossible to infer which databases were searched, or if the search terms were applied across a range of databases.

When giving advice on designing a search strategy, the Cochrane handbook conveys the following summary point: “Avoid too many different search concepts but use a wide variety of synonyms and related terms (both free text and controlled vocabulary terms) combined with ‘OR’ within each concept” (Higgins and Green, 2011). Only four publications in our sample use subject headings. This corresponds with the findings from a study of 300 systematic reviews, where 12% reported using controlled vocabulary terms (Page et al., 2016). It can be questioned whether authors are aware of the increased needs for accuracy and comprehensiveness in the search strategy of a systematic review or meta-analysis and the importance of using subject headings (Sampson et al., 2009; McGowan et al., 2016; Salvador-Olivan et al., 2019), or if they should seek assistance for designing the search strategy. Table 6 illustrates how different text words are interpreted by different databases, and the consequent need for adaptation of search strategies.

**TABLE 6:** Subject specific example of text word searching in two databases.

**Data base/Text word search**	**Post-traumatic**	**Post traumatic**	**Posttraumatic**	**post?traumatic**
PsycInfo (OVID)^1^	14907	14907	42187	42187
PubMed^2^	52015	61763	33902	52015

*Number of results from text word search in default fields. ^1^Text word.mp. ^2^Text word searched in All Fields.*

Research librarians are methodologists and can be included as part of teams conducting reviews (Rethlefsen et al., 2015; Metzendorf and Featherstone, 2018), or librarians can peer review search strategies for systematic reviews, similar to statistics experts (Koffel and Rethlefsen, 2016). The PRESS-guidelines assume that a librarian or an information specialist plans, performs, and reviews a search strategy (Sampson et al., 2009; McGowan et al., 2016). This can have some advantages for the review team, namely time saved through improved search precision, resulting in less studies to examine, and avoidance of late adjustments to the search strategy, and consequently more studies to examine (Sampson et al., 2009). Teams conducting systematic reviews could profit from an inter-disciplinary approach, involving research librarians. We would welcome a discussion on the inclusion of subject terms as a specific criterion for critical appraisal in AMSTAR 2 (Shea et al., 2017), and a more specific reference in item 8 of the PRISMA statement (Liberati et al., 2009).

As far as we can tell, the search criteria stated as a minimum by the Cochrane handbook (Higgins and Green, 2011) are manageable for authors. The discussion on methodology for systematic reviews, and whether we should differentiate between elaborate reviews and ‘leaner’ reviews to cover important clinical questions, is essential (Bastian et al., 2010).

Readers often use titles of publications as a first sifting instrument, thus the title should describe the content of the articles precisely. The PRISMA-guidelines advise including information about the participants, intervention, comparator, outcome, and study design in the title (Liberati et al., 2009). Seventeen of the twenty reviews in our sample included the term ‘systematic review’ or ‘meta-analysis’ in the title. Of the three that failed to do so, one provided this information in the abstract, and the other two in the full text. Page et al. (2016) found that 94% of the publications examined included “systematic review” or “meta-analysis” in the title, increasing from 68.2% for non-Cochrane reviews in 2004 (Moher et al., 2007). Another new study using the PRISMA to assess quality, found that 79 % of systematic reviews include this information (Sharma and Oremus, 2018). We expect a further rise in precise titles, as found by Page, and believe readers will welcome this improvement.

Nine out of the twenty reviews in our sample provided general information on the time span of their search, and twelve out of twenty stated the date on which the search ended. According to the PRISMA-guidelines, information on dates of coverage should be given for each database (Liberati et al., 2009). It is important to distinguish between coverage for each database and time span as general information (Koffel and Rethlefsen, 2016), as databases such as Medline, Embase, and PsycInfo can be searched for different timespans. Page et al. (2016) report that 65% of studies contain information on start and end dates for all databases, while for Cochrane reviews the share is 91%, and in their sample, 29% of the reviews examined gave general information on time span. Koffel and Rethlefsen (2016) found that 25% of the systematic reviews provide start and end dates for each database. Our sample has only general information on included time span for the search, and in addition, we observed a lack of specific information on database providers. This may signal a need for authors to be more conscientious in reporting their methods. Information on timespan for the databases and last date of the search is necessary to reproduce the search strategy and for updating reviews (Liberati et al., 2009).

The majority of reviews we examined added supplementary techniques to their search strategy to support the collection of relevant studies. The most commonly used technique found in other studies was to review reference lists (Koffel and Rethlefsen, 2016; Page et al., 2016), while other techniques can be identifying unpublished trials, or contacts with experts. Among other things, the aim of using supplementary techniques is to reduce the risk of missing relevant studies, and publication bias (Cooper et al., 2018).

The PRISMA guidelines advise that the authors of systematic reviews should include a full search strategy for at least one database (Liberati et al., 2009). Only five of the twenty reviews or meta-analyses in our sample fulfilled this criterion. This is similar to findings by Koffel and Rethlefsen (2016) [24%] and Page et al. (2016) [30%] for non-Cochrane reviews, while Sharma and Oremus (2018) report higher adherence [63.2%]. The main reason for this advice is to make critical evaluation possible and allow replication and updating (Bastian et al., 2010; McGowan et al., 2016), thus giving the reader a chance to decide whether or not the foundations of the work are sound (Sampson et al., 2009).

Similar to Koffel and Rethlefsen (2016), we find that a majority of reviews report their search strategies at a level that is too general, hence insufficient for replication. Item 7 of the PRISMA guideline recommends detailed reporting of information sources, whereas Item 8, Search, can be answered with Yes or No. This can explain why Sharma and Oremus (2018) found high compliance with Item 7 in their study. We argue that adherence to the PRISMA guidelines is necessary to provide sufficient detail to reproduce and assess search strategies.

Flow charts to illustrate identification, screening, eligibility, and inclusion, are used in 14 of the 20 [70%] reviews in our study, although only three of these use the PRISMA flow diagram (Moher et al., 2009). This result corresponds with Page et al. (2016) and Sharma and Oremus (Sharma and Oremus, 2018), 69 and 89.5% respectively. Page et al. differentiate between complete, partial, and no reporting, which can explain the difference.

In their study of systematic reviews from 2012, Koffel and Rethlefsen (2016) ask if enhanced knowledge of reporting and methodology guidelines will improve reporting quality. Our study of systematic reviews and meta-analyses published between 2011 and 2017, using PRISMA and Cochrane Handbook criteria shows no positive development regarding reporting quality. Although some authors, [3/20] in our sample, stated that they followed the PRISMA guidelines, we found that they did not comply with them (Liberati et al., 2009). One explanation can be that editors are more occupied with other details, or lack resources to follow-up on reporting or methodology standards (Moher et al., 2007). Other reasons might be the lack of courses for researchers on systematic search methods and reporting standards (Koffel and Rethlefsen, 2016), or the lack of software to help authors report their systematic review (Page et al., 2016). For the time being, PRISMA checklists do not ensure actual use of the guidelines (Page et al., 2016), and can appear to be a “ticking-the-boxes” exercise (Greenhalgh et al., 2018).

As we noted a large number of reviews and meta-analyses on PTSD in adults in our initial scoping search, we added an extra criterion assessing whether the publications referred to a specific Cochrane review (Bisson and Andrew, 2007; Bisson et al., 2013) addressing this research question. Interestingly, seven of the twenty reviews do not refer to the Bisson review (Bisson and Andrew, 2007; Bisson et al., 2013). It is important that authors of reviews or meta-analyses place their work in the context of other reviews (Bastian et al., 2010). Furthermore, Bastian et al. (2010) point out that this is also the responsibility of editors.

In general, the studies identified in a systematic search form the basis of the review process, and should build a sound base for analyzing and synthesizing evidence (Cooper et al., 2018), and minimize bias in forming conclusions. If the methods used for searching are not systematic or the search strategy is flawed, there is a risk of not including all relevant studies, thus influencing the conclusions of systematic reviews (Salvador-Olivan et al., 2019).

More specifically, poor systematic reviews can affect the field of psychotherapy, and treatments such as EMDR, negatively, as mental health practitioners who wish to find evidence based treatments they can use to treat their patients, may become confused by conflicting statements.

In the present paper, we find that half of the reviews have a score of three points or lower. This result suggests that there is not enough attention to the literature search process. The different phases of planning and doing a systematic review have their own methodological challenges, and may introduce bias in conclusions. There is a possibility that poor quality in the reviews can lead to inaccurate conclusions. We have not focused directly on the conclusions from each of the reviews. That may become the subject of another paper.

There are some weaknesses in this study. Our examination of search methods relies on reported characteristics, and our sample is small. However, assessing systematic reviews and meta-analyses in light of a relatively narrow research question reveals results that raise some important issues regarding the trustworthiness of the body of knowledge pertaining to that question. Furthermore, two reviewers individually screened the methods sections of the reviews and assessed the overall confidence in the completeness of the search. In our evaluation we used the minimum criteria in the Cochrane Handbook (Higgins and Green, 2011) and PRISMA (Liberati et al., 2009) for systematic reviews and meta-analyses, and have discussed our findings with reference to studies with larger samples.

## Conclusion

The aim of this article has been to evaluate the quality of non-Cochrane systematic reviews addressing the subject of EMDR as a therapy commonly used for PTSD by analyzing their search and reporting methods. We found inadequacies in the methods employed for searching and reporting the search strategy, which could have been avoided by greater adherence to guiding documents for performing systematic reviews.

The methods used for performing and reporting a search can, like other methods used in a systematic review, enhance or undermine trustworthiness. Review teams could profit from involving different methodologists, such as research librarians. Alternatively, authors should look for, and participate in, courses on systematic searching. Table 7 lists some key points relevant for authors of systematic reviews.

**TABLE 7:** Good practice key points for effective searching and reporting.

• Identify existing systematic reviews that are relevant to your question to avoid redundancy
• Include a librarian with expertise in searching in your team when planning search strategies for relevant databases
• Make yourself familiar with central resources for systematic searching methodology
• Carefully read and adhere to the explanation and elaboration document for PRISMA (Liberati et al., 2009)

Our findings raise important questions for future debate on the risk of omitting studies, thus impairing the conclusions of a systematic review. For clinical purposes, researchers should investigate if, and how, the search strategy in a systematic review affects the body of knowledge and the results. Poor systematic reviews may confuse mental health practitioners who wish to find evidence-based treatments they can use to treat their patients.

## Data Availability

The datasets generated for this study are available on request to the corresponding author.

## Author Contributions

EO and PA appraised included studies. EO drafted the manuscript. PA, MJ, BA, and KK contributed to critical revision of the draft. All authors conceived and designed the study, and read and approved the final version of the manuscript.

## Conflict of Interest Statement

The authors declare that the research was conducted in the absence of any commercial or financial relationships that could be construed as a potential conflict of interest.

## Footnotes

1 https://www.ncbi.nlm.nih.gov/mesh
